# A Hybrid Low-Complexity WMMSE Precoder with Adaptive Damping for Massive Multi-User Multiple-Input Multiple- Output Systems

**DOI:** 10.3390/s25226827

**Published:** 2025-11-07

**Authors:** Vaskar Sen, Honggui Deng, Xiaowen Xu, Menghui Shen

**Affiliations:** School of Electronic Information, Central South University, Changsha 410004, China; vaskarsen@csu.edu.cn (V.S.); xuxiaowen@csu.edu.cn (X.X.); 244512039@csu.edu.cn (M.S.)

**Keywords:** low-complexity precoding, weighted minimum mean square error (WMMSE), massive MU-MIMO, adaptive damping, hybrid switching

## Abstract

Maximizing the weighted sum-rate (WSR) in downlink multi-user multiple-input multipleoutput (MU-MIMO) systems remains computationally challenging due to the prohibitive complexity of classical weighted minimum mean square error (WMMSE) algorithms. In this article, we propose a novel low-complexity WMMSE (LC-WMMSE) precoding method specifically designed for massive MU-MIMO downlink systems. Our algorithm introduces a hybrid switching approach that adaptively blends standard WMMSE updates with computationally simpler approximations derived via the Woodbury matrix identity, coupled with an adaptive damping mechanism to ensure robust and stable convergence. Simulation results demonstrate that the proposed LC-WMMSE method achieves WSR performance comparable to classical WMMSE but with significantly reduced computational complexity, making it particularly suitable for practical implementation for massive MUMIMO systems.

## 1. Introduction

Massive MU-MIMO systems are one of the key enabling technologies for fifthgeneration (5G) and next-generation wireless communication networks, owing to their capability to substantially enhance spectral efficiency, reliability, and network capacity [1,2,3,4]. In multi-user (MU) scenarios, effectively managing inter-user interference through optimal precoding is essential to fully exploit these advantages. Among existing precoding methods, the weighted minimum mean square error (WMMSE) algorithm is widely recognized for delivering near-optimal weighted sum-rate (WSR) maximization in practical MU-MIMO systems [5,6,7]. However, the WMMSE algorithm involves multiple high-dimensional matrix inversions within each iteration, resulting in a computational complexity that scales cubically with the number of base station antennas [8,9]. Such complexity severely restricts the practical feasibility of classical WMMSE for large-scale antenna arrays typically found in massive MU-MIMO deployments. To overcome these limitations, recent research has focused on low-complexity alternatives that approximate the WMMSE performance with minimal loss in optimality. Most existing approaches, however, rely on fixed approximations or simplified update rules [10,11], often resulting in noticeable performance degradation and convergence behavior in large-scale scenarios. The challenge of maximizing the weighted sum rate (WSR) problem in the downlink under a sum power constraint (SPC) is non-convex and known to be (non-deterministic polynomial-time hardness) NPhard [10,12,13]. R-WMMSE [10] computational cost through randomized sketching (data reduction), whereas LC-WMMSE reduces cost via structure exploitation, a Woodbury reformulation with a diagonal-weight surrogate in the transmit update step. The former incurs probabilistic approximation from sketching; the latter is deterministic with complexity mainly tied to the stream dimension rather than the number of base station (BS) antennas. Consequently, our study focused on developing practical, high-performance precoders with manageable computational complexity. Global solutions thus typically involve exponential computational complexity, rendering them impractical for massive MU-MIMO systems. Non-iterative methods, such as maximum ratio transmission (MRT) [14], zero-forcing (ZF) [15], and regularized ZF (RZF) [16] precoding, which offer closed-form solutions with computational efficiency, significantly compromise WSR performance due to their inability to directly optimize the WSR objective. Iterative algorithms for WSR maximization are mainly divided into two categories, one of which is the successive convex approximation (SCA) method. In this approach, the authors [17,18] convex surrogate problems of the non-convex WSR objective and solved the convex problem to increase the WSR, with proven convergence to a stationary point, and various extensions have been proposed to handle different system scenarios [19,20]. The other major class of iterative precoding algorithms is the classical weighted minimum mean-square error (WMMSE) method [21], which exploits the fundamental relationship between the mean-square error (MSE) and the signal-to-interference-plus-noise ratio (SINR). By iteratively minimizing the weighted MSE problem, which is iteratively solved by applying the block coordinate descent (BCD) method [22], this leads to the WMMSE algorithm with three closed-form updates. The WMMSE algorithm updates are derived using the BCD method, ensuring efficient convergence to a stationary point of the WSR maximization problem. Nonetheless, most of these approaches either suffer from noticeable performance degradation or fail to ensure stable convergence in large-scale scenarios. Among these works, in the context of uplink detection, the adaptive damped Jacobi (DJ) method [23] has been proposed to iteratively approximate the MMSE problem solution. The author introduced an adaptive damping Jacobi method that dynamically updates the optimal relaxation factor ω with the increase in iterations performance automatically and particularly in correlated channels. This demonstrates the increasing use of adaptive damping techniques for stabilizing and accelerating iterative algorithms for massive MIMO systems. However, using such an adaptive method for the downlink precoding problem remains underexplored. The weighted sum rate (WSR) maximization problem for precoding presents a different set of challenges, with a different system model and a non-convex objective function. Motivated by these challenges, in this paper we propose a novel low-complexity WMMSE (LC-WMMSE) precoding algorithm tailored explicitly for massive MU-MIMO downlink systems. The key innovations of our approach are twofold: First, we introduce a hybrid switching technique, which adaptively combines computationally intensive classical WMMSE updates with lightweight approximations via an adaptive mixing parameter, thereby significantly reducing complexity during initial iterations without compromising the final WSR performance. Second, we integrate an adaptive damping mechanism, which stabilizes precoder updates and ensures robust and reliable convergence behavior throughout iterations of the iterative optimization process. In summary, our primary contributions in this paper are as follows:

- We propose a novel low-complexity WMMSE (LC-WMMSE) precoding algorithm that employs the Woodbury identity to avoid large matrix inversions, significantly reducing computational complexity while maintaining near-optimal performance.
- We introduce a hybrid switching $\omega^{\left(t\right)}$ technique that dynamically blends full WMMSE precoder updates with lightweight approximations via an adaptive mixing factor $\alpha^{\left(t\right)}$. This approach strategically reduces computational complexity during initial iterations without compromising the final weighted sum-rate (WSR) performance.
- To guarantee monotonic improvement of the WSR objective, we integrate an adaptive damping mechanism into the precoder update procedure. This adaptive strategy significantly enhances convergence stability and robustness, which is beneficial in large-scale system deployments.
- We derive closed-form update rules for all core components of the precoding framework. Specifically, receive filters, weight matrices, and precoders, facilitating efficient practical implementation and reducing computational overhead.
- Through comprehensive simulations, we demonstrate that our proposed LC-WMMSE algorithm achieves near-identical WSR performance to the classical WMMSE algorithm while substantially reducing computational runtime. Unlike existing low-complexity methods, our algorithm uniquely combines adaptive damping and hybrid switching, resulting in superior convergence reliability and efficiency, particularly suited for massive MU-MIMO deployments.

Table 1 summarizes the MIMO research areas that are the focus of the contributions from the previously cited works. R-WMMSE [10] reduces computational cost by solving the WMMSE normal equations in a compressed domain through randomized sketching, which effectively performs data dimensionality reduction. In contrast, LC-WMMSE retains the full channel representation and achieves cost efficiency through structural exploitation—specifically, by employing a Woodbury matrix identity reformulation and utilizing a diagonal-weight surrogate exclusively during the transmit filter update. Consequently, the two methods differ fundamentally in terms of update dimensionality, dominant computational complexity, and the source of approximation.

The remainder of this paper is organized as follows. Section 2 describes the system model and problem formulation. Section 3 proposed LC-WMMSE, including detailed derivations and complexity analysis. Simulation results are presented and discussed in Section 4. Finally, conclusions are drawn in Section 5.

## 2. System Model

### 2.1. Downlink System Model

We consider a single-cell downlink MU-MIMO system where a base station (BS) with *M* transmit antennas serves *K* users, each equipped with *N* receive antennas. The downlink channel from the BS to the user *k* is

(1) $$H_{k}\in C^{M\times N},\;k=1,\ldots ,K,$$

whose entries are modeled as i.i.d. circularly symmetric complex Gaussian random variables with zero mean and unit variance, i.e., Rayleigh fading. The BS transmits the signal

(2) $$x=\sum_{k=1}^{K}P_{k}s_{k}\in C^{M\times 1},$$

where $P_{k}\in C^{M\times d_{k}}$ is the linear precoder for the user *k*, and $s_{k}\in C^{d_{k}\times 1}$ is the data symbol vector for the user *k* with $E\left[s_{k}s_{k}^{H}\right]=I_{N}$.

Under a flat-fading assumption, the received signal at the user *k* is

(3) $$y_{k}=H_{k}x+n_{k}=H_{k}\sum_{j=1}^{K}P_{j}s_{j}+n_{k},$$

where $n_{k}∼\mathrm{CN}\left(0,\sigma^{2}I_{N}\right)$ is additive white Gaussian noise. The data vectors $\left\{s_{k}\right\}$ are mutually independent and independent of $\left\{n_{k}\right\}$. All symbols used in this paper are summarized in Table 2.

**Remark** **1.** *(Scalability in Massive MIMO): In practical massive MU-MIMO systems, the number of antennas at the base station (BS) is significantly larger than the number of antennas at each user [24], and the number of users, i.e., we have M≫K≥N. In such cases, classical algorithms like WMMSE involve large matrix inversions and thus suffer from high computational complexity that scales poorly with M. To address this, the proposed LC-WMMSE algorithm incorporates hybrid switching and adaptive damping techniques, which substantially reduce the complexity. These techniques allow the algorithm to scale efficiently with the number of BS antennas, achieving complexity that is approximately sub-cubic independent of M in large-scale settings.*

### 2.2. Problem Formulation

A fundamental objective in downlink MU–MIMO is to design the precoders ${\left\{P_{k}\right\}}_{k=1}^{K}$ to maximize the weighted sum rate (WSR) subject to a transmit power constraint. Let $\mu_{k}\geq 0$ denote the weight for user *k*. The WSR defined as

(4) $$R=\sum_{k=1}^{K}\mu_{k}R_{k},$$

where the achievable rate of user *k* is

(5) $$R_{k}=\log_{2}\det\;\left(I_{N}+\Sigma_{k}^{-1}\;H_{k}P_{k}P_{k}^{H}H_{k}^{H}\right),$$

where the covariance matrix of interference-plus-noise given by

(6) $$\Sigma_{k}=\sigma^{2}I_{N}+\sum_{\begin{array}{c} j=1\\ j\neq k \end{array}}^{K}H_{k}P_{j}P_{j}^{H}H_{k}^{H}.$$

The optimization problem is to maximize the WSR over all feasible precoders under either a sum power constraint (SPC). These constraints yield different formulations and trade-offs in performance and complexity.

Under the sum power constraint (SPC), the WSR maximization problem can be formulated as

(7) $$\begin{array}{cc} \max_{\left\{P_{k}\right\}}\; & \sum_{k=1}^{K}\mu_{k}R_{k},\\ s.t.\; & \sum_{k=1}^{K}\mathrm{tr}\;\left(P_{k}P_{k}^{H}\right)\leq P_{\max}, \end{array}$$

where $P_{\max}$ represents the total transmit power budget of BS. The WSR maximization problem formulated in Equation (7) is challenging due to the highly nonlinear and non-convex nature of the WSR objective function. Moreover, following [13], it can be shown that both problems are NP-hard, as stated in the following proposition.

**Proposition** **1.** *(WSR maximization is NP-hard): Equation (7) is NP-hard under sum power constraints.*

## 3. Proposed LC-WMMSE Algorithm

### 3.1. The Classical WMMSE Reformulation

The WMMSE framework is mostly used for WSR maximization problems [10]. In this section, we revisit the classical WMMSE approach [21,25] from a purely optimizationtheoretic viewpoint, where the mean square error (MSE) does not require a physically meaningful interpretation. The WSR maximization problem in Equation (7) is non-convex and difficult to solve directly. Using the equivalence between rate maximization and weighted MSE minimization, which can be solved by the BCD method [21], the problem can be reformulated as

(8) $$\begin{array}{cc} \min_{\left\{W_{k},U_{k},P_{k}\right\}}\; & \;\sum_{k=1}^{K}\mu_{k}\left(\mathrm{Tr}\left(W_{k}E_{k}\right)-\log\det\left(W_{k}\right)\right)\\ s.t.\;\; & \sum_{k=1}^{K}{∥P_{k}∥}_{F}^{2}\leq P_{\max} \end{array}$$

Subject to the same transmit power constraint as in Equation (8). Here, $\mu_{k}\geq 0$ is the priority weight for the user *k*, and the MSE matrix $E_{k}$ for the user *k* is defined by

(9) $$E_{k}\;=\;E\;\left[\;\left(U_{k}^{H}y_{k}-s_{k}\right)\;{\left(U_{k}^{H}y_{k}-s_{k}\right)}^{H}\right].$$

where the mean square error (MSE) matrix for the user *k*, $U_{k}\in C^{N\times d_{k}}$ is the receive filter and $W_{k}\in C^{d_{k}\times d_{k}}$ is the weight matrix, both to be optimized jointly with the precoders $P_{k}$. Expanding the MSE matrix $E_{k}$,

(10) $$\begin{array}{cc} E_{k} & =I_{N}-U_{k}^{H}H_{k}P_{k}-P_{k}^{H}H_{k}U_{k}\\ \; & \;+\sum_{j=1}^{K}U_{k}^{H}H_{k}P_{j}P_{j}^{H}H_{k}^{H}U_{k}+\sigma^{2}U_{k}^{H}U_{k}. \end{array}$$

The reformulated objective in Equation (8) is jointly non-convex in $\left\{P_{k},U_{k},W_{k}\right\}$, but is convex in each variable individually. Therefore, the optimization can be solved using an alternating optimization approach as follows:

(11) $$U_{k}={\left(\sum_{j=1}^{K}H_{k}P_{j}P_{j}^{H}H_{k}^{H}+\sigma^{2}I_{N}\right)}^{-1}H_{k}P_{k},\;\forall k.$$

The update of $W_{k}$ while fixing the other two block variables is given by

(12) $$\begin{array}{c} W_{k}=\mu_{k}{\left(E_{k}+\varepsilon \;I_{d_{k}}\right)}^{-1},\;E_{k}\in C^{d_{k}\times d_{k}},\;\forall k. \end{array}$$

While fixing $U_{k}$ and $W_{k}$, the precoder update is obtained by solving the following problem

(13) $$\begin{array}{cc} A & ≜\sigma^{2}I_{M}+\sum_{k=1}^{K}H_{k}U_{k}W_{k}U_{k}^{H}H_{k}^{H}\in C^{M\times M}, \end{array}$$

(14) $$\begin{array}{cc} B & ≜\left[\;H_{1}U_{1}W_{1}\;\ldots \;H_{K}U_{K}W_{K}\;\right]\in C^{M\times D},\;D=\sum_{k=1}^{K}d_{k}. \end{array}$$

where, $A\in C^{M\times M},\;B\in C^{M\times D},\;P\in C^{M\times D},\;P_{k}\in C^{M\times d_{k}}$. We solve the linear system AP=B for P; equivalently,

(15) $$P=A^{-1}B\;\;⟺\;\;P_{k}=A^{-1}\left(H_{k}U_{k}W_{k}\right),\;k=1,\ldots ,K.$$

Although the same A is used for all users, the right-hand blocks $H_{k}U_{k}W_{k}$ differ; hence, the results $P_{k}$ are user-specific. Since $\sigma^{2}>0$ and ε>0 in Equation (12), A is Hermitian positive definite, the system is well posed.

(16) $$s.t.\;P\leftarrow \sqrt{\frac{P_{\max}}{\max(||P|{|}_{F}^{2},\epsilon )}}\cdot P.$$

Here, $P_{max}$ is the total transmit power and ϵ>0 is a small regularization constant (e.g., $10^{-8}$) for numerical stability. For the LC update, we replace $W_{k}$ by $D_{k}=\mathrm{diag}\left(W_{k}\right)$ in Equation (13), Equation (14) and compute P via the Woodbury identity to avoid the M×M inversion.

Although the classical WMMSE precoding algorithm involves multiple large-scale matrix inversions at each iteration, each of size M×M. Thus, the computational complexity is dominated by these inversions, resulting in a prohibitive cubic complexity of $O(M^{3})$. This complexity becomes particularly challenging in massive MU-MIMO scenarios where *M* is very large. Thus, each iteration requires cubic operations, severely limiting scalability in massive MU-MIMO deployments. This motivates the need for efficient alternatives that reduce matrix inversion cost, as addressed in our proposed LC-WMMSE framework in the next subsection.

### 3.2. Proposed LC-WMMSE

In this subsection, as we mentioned in Section 3.1, the original WMMSE algorithm for the SPC case in [21] requires a high-dimensional matrix operation at each iteration. Motivated by the prohibitive cubic complexity, we propose a novel LC-WMMSE precoding method designed explicitly to reduce computational complexity significantly while maintaining near-optimal performance for massive MU-MIMO systems. Our method integrates hybrid switching, adaptive damping, and simplified precoding approximations to significantly reduce computational complexity while maintaining robust convergence and high performance.

#### 3.2.1. Problem Reformulation

Our LC-WMMSE replaces the M×M inversion in Equations (13)–(15) by a (NK)×(NK) solve via Woodbury, cutting the dominant per-iteration cost from $O(M^{3})$ to $O\left(M{\left(NK\right)}^{2}\right)+{\left(NK\right)}^{3})$ in the massive MIMO regime M≫NK as follows:

- Hybrid Transmit Precoder Update: The transmit precoder update at each iteration is computed using a hybrid combination of the classical WMMSE precoder $P_{\mathrm{WMMSE}}^{\left(t\right)}$ and a low-complexity approximation precoder $P_{\mathrm{LC}}^{\left(t\right)}$ as follows:
  (17) $$P^{\left(t\right)}=\omega^{\left(t\right)}P_{\mathrm{WMMSE}}^{\left(t\right)}+\left(1-\omega^{\left(t\right)}\right)P_{\mathrm{LC}}^{\left(t\right)}.$$
  where $P_{\mathrm{WMMSE}}^{\left(t\right)}$ is the classical precoder from Equation (15), $P_{\mathrm{LC}}^{\left(t\right)}$ is a low-complexity approximation precoder computed with simplified operations to avoid costly matrix inversions and $\omega^{\left(t\right)}\in \left[0,1\right]$ is an adaptive switching factor designed to balance accuracy and computational efficiency. Specifically, we define $\omega^{\left(t\right)}$ as
  (18) $$\omega^{\left(t\right)}=\frac{∥\Delta E^{\left(t\right)}{∥}_{F}}{∥\Delta E^{\left(t\right)}{∥}_{F}+\kappa }$$
  where
  (19) $$\Delta E^{\left(t\right)}=\sum_{k=1}^{K}E_{k}^{\left(t\right)}-E_{k}^{\left(t-1\right)}$$
  The factor $\omega^{\left(t\right)}\;\in \left[0,1\right]$ in Equations (18) and (19) measures how much the per-iteration MSE changes: When $∥\Delta E^{\left(t\right)}{∥}_{F}$ is large (the algorithm is far from a fixed point), $\omega^{\left(t\right)}\;\approx \;1$, we favor the accurate WMMSE update; near convergence $∥\Delta E^{\left(t\right)}{∥}_{F}$ is small, so $\omega^{\left(t\right)}\;\approx \;0$, we favor the low-complexity step to save computation. This approach of monitoring convergence progress to guide algorithmic behavior follows established optimization principles [22]. The constant κ>0 smooths the ratio and prevents division by zero (we use $\kappa =10^{-3}$ unless otherwise stated). In our experiments, results are insensitive to $\kappa \in [10^{-4},10^{-2}]$ (final WSR variation <0.3%). The weight as
  (20) $$D_{k}^{\left(t\right)}=\mathrm{diag}\;\left(\mathrm{diag}(W_{k}^{\left(t\right)})\right)≻0,\;D_{k}^{\left(t\right)}\in C^{d_{k}\times d_{k}},\;k=1,\ldots ,K.$$
  We approximate the full weight by its diagonal $D_{k}^{\left(t\right)}=\mathrm{diag}\;\left(\mathrm{diag}(W_{k}^{\left(t\right)})\right)\in C^{d_{k}\times d_{k}}$, which preserves positive definiteness (diagonal entries are strictly positive due to the regularized MSE) while removing inter-stream couplings. This diagonal form is key to building the block-diagonal matrix in Equation (21), enabling a smaller inversion in the Woodbury step. Using $U_{k}^{\left(t\right)}$ and $D_{k}^{\left(t\right)}$, we set
  (21) $$S^{\left(t\right)}=\mathrm{blkdiag}\;\left(U_{1}^{\left(t\right)}D_{1}^{\left(t\right)}{U_{1}^{\left(t\right)}}^{H},\ldots ,U_{K}^{\left(t\right)}D_{K}^{\left(t\right)}{U_{K}^{\left(t\right)}}^{H}\right)\in C^{\left(NK)\times (NK\right)},$$
  Each block $U_{k}^{\left(t\right)}D_{k}^{\left(t\right)}{U_{k}^{\left(t\right)}}^{H}$ is Hermitian positive definite; therefore $S^{\left(t\right)}≻0$. In the LC update, the inverse of $S^{\left(t\right)}$ appears inside a (NK)×(NK) inversion, so the cubic term scales with NK rather than *M*. We horizontally stack the user channels as
  (22) $$H=\left[\;H_{1},\ldots ,H_{K}\;\right]\in C^{M\times (NK)},$$
  This makes the normal matrix $\sigma^{2}I_{M}+HS^{\left(t\right)}H^{H}$ compact and enables the Woodbury identity to trade an M×M inversion for a (NK)×(NK) inversion. The right-hand factor for the precoder update,
  (23) $$B^{\left(t\right)}=\left[\;H_{1}U_{1}^{\left(t\right)}D_{1}^{\left(t\right)},\ldots ,H_{K}U_{K}^{\left(t\right)}D_{K}^{\left(t\right)}\;\right]\in C^{M\times D}.$$
  Here $B^{\left(t\right)}$ concatenates the per-user factors; the *k*-th block column generates $P_{k}^{\left(t\right)}$. With the stacked form, the Woodbury precoder Equation (32) returns $P^{\left(t\right)}\in C^{M\times D}$ whose *k*-th column block is the user precoder $P_{k}^{\left(t\right)}$. Forming $H^{H}B^{\left(t\right)}$ costs O(M·NK·D), followed by a (NK)×(NK) SPD inversion—much cheaper than an M×M inversion when NK≪M. Similarly to prior works [10,21], we apply global power normalization at each iteration to ensure the total transmit power constraint is satisfied. After updating the precoders, we scale them uniformly as follows:
  (24) $$P_{k}^{\left(t\right)}\;\leftarrow \;\sqrt{\frac{P_{max}}{\sum_{k=1}^{K}{∥P_{k}^{\left(t\right)}∥}_{F}^{2}}}\;P_{k}^{\left(t\right)},\;\forall k,$$
  where $P_{max}$ is the total transmit power budget of BS. This approach simplifies implementation and preserves convergence, leveraging the fact that the WSR objective is invariant to common scaling of the precoders.
  We simplify the computationally intensive classical WMMSE precoder update by approximating the involved matrix inversions. Specifically, the proposed hybrid switching approach significantly reduces the frequency of expensive matrix inversions during the iterative procedure. Furthermore, the simplified low-complexity approximation in Equations (20)–(23) employs diagonal approximations and diagonal loading instead of a full M×M matrix inversion, thus reducing complexity from cubic.
- Adaptive Damping Factor: To ensure stable and monotonic convergence, we adapt the damping as
  (25) $$\Delta R^{\left(t\right)}≜|{WSR}^{\left(t\right)}-{WSR}^{\left(t-1\right)}|,\;\alpha^{\left(t\right)}=\mathrm{clip}\;\left(1-\frac{\Delta R^{\left(t\right)}}{\eta },\;\alpha_{\min},\;\alpha_{\max}\right),$$
  The adaptive damping $\alpha^{\left(t\right)}=\mathrm{clip}\;\left(1-\Delta R^{\left(t\right)}/\eta ,\;\alpha_{\min},\alpha_{\max}\right)$ reduces the step size when the WSR varies rapidly (large $\Delta R^{\left(t\right)}$), which stabilizes the iterates without sacrificing monotonic ascent; when changes are small, it allows larger updates for faster progress. Unless otherwise stated, we use $\eta =10^{-3}$, $\alpha_{\min}=0.2$, and $\alpha_{\max}=0.9$ in all experiments, and we apply a short Armijo backtracking (up to 5 trials) to ensure $\mathrm{WSR}\;\left(P^{\left(t+1\right)}\right)\geq \mathrm{WSR}\;\left(P^{\left(t\right)}\right)$. Sensitivity tests showed the results are robust for $\eta \in [10^{-4},10^{-2}]$. The smoothed precoder update is
  (26) $$P^{\left(t\right)}\leftarrow \alpha^{\left(t\right)}P^{\left(t-1\right)}+\left(1-\alpha^{\left(t\right)}\right)\;{\hat{P}}^{\left(t\right)},$$
  where ${\hat{P}}^{\left(t\right)}$ is the current LC-WMMSE update before damping. We apply a short Armijo backtracking on $\alpha^{\left(t\right)}$ (at most 5 trials) and accept the first $\alpha^{\left(t\right)}$ such that $WSR\;\left(\alpha^{\left(t\right)}P^{\left(t-1\right)}+\left(1-\alpha^{\left(t\right)}\right){\hat{P}}^{\left(t\right)}\right)\geq WSR\;\left(P^{\left(t-1\right)}\right)$. This stabilizes the iterates and typically does not increase runtime. The adaptive damping mechanism dynamically adjusts the update steps based on the rate of improvement at each iteration, ensuring stable convergence. At iteration *t*, the instantaneous WSR achieved by the proposed LC-WMMSE algorithm is given by
  (27) $${WSR}^{\left(t\right)}=\sum_{k=1}^{K}\mu_{k}\log_{2}\det\left(I_{N}+H_{k}P_{k}^{\left(t\right)}P_{k}^{(t)H}H_{k}^{H}{\left(\sigma^{2}I_{N}+\sum_{j\neq k}H_{k}P_{j}^{\left(t\right)}P_{j}^{(t)H}H_{k}^{H}\right)}^{-1}\right)$$
  (28) $$s.t.\;\sum_{k=1}^{K}{∥P_{k}∥}_{F}^{2}=P_{\max}$$
  which respects the total power budget at the transmitter. $\left\{P_{k}^{\left(t\right)}\right\}$ denotes the precoders updated at iteration *t*. This metric is used to monitor convergence and evaluate performance.

#### 3.2.2. Adaptive Damping Mechanism

**Figure 1:**
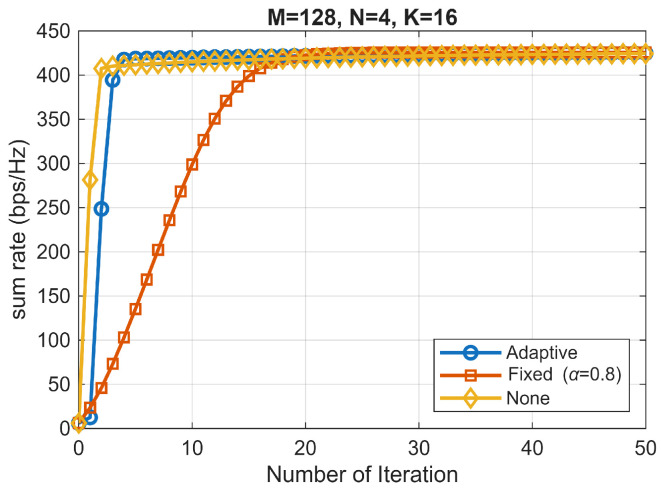
WSR vs. iteration at 20 dB (i.i.d. Rayleigh), averaged over 100 trials. Final WSRs are nearly identical (Adaptive 424.90±2.01, Fixed 425.79±2.03, None 424.42±2.12). Adaptive reaches the plateau in a few iterations, whereas Fixed converges slowly, and None shows larger early overshoots.

Figure 1 compares LC-WMMSE with adaptive damping, Fixed damping (α=0.8), and None (α≡1) at M=128, K=16, N=4 and SNR 20 dB (mean over 100 trials). In Table 3 all variants reach essentially the same final WSR (Adaptive 424.90±2.01, Fixed 425.79±2.03, None 424.42±2.12), but adaptive attains the plateau in far fewer iterations and exhibits smaller late-iteration oscillations. This confirms that adaptive damping improves convergence speed and stability without degrading WSR.

The oscillation index quantifies the variance observed over the most recent 10 iterations, with lower values indicating greater convergence stability.

### 3.3. Proposed LC-WMMSE Updates Precoder

The proposed low-complexity WMMSE (LC-WMMSE) precoding algorithm is summarized in Algorithm 1 and consists of the following three main steps:

- Receive Filter Update $U_{k}^{\left(t\right)}$: At iteration *t* the receive filter for user *k* is updated as
  (29) $$U_{k}^{\left(t\right)}={\left(H_{k}^{H}\;S_{x}^{\left(t\;-\;1\right)}\;H_{k}+\sigma^{2}I_{N}\right)}^{-1}H_{k}^{H}\;P_{k}^{\left(t\;-\;1\right)},\;\forall k.$$
  Here $S_{x}^{\left(t\;-\;1\right)}$ is the BS transmit covariance formed from the precoders at the previous iteration:
  (30) $$S_{x}^{\left(t\;-\;1\right)}=\sum_{j=1}^{K}P_{j}^{\left(t\;-\;1\right)}\;P_{j}^{{\left(t\;-\;1\right)}^{H}}\in C^{M\times M}.$$
  The term $H_{k}^{H}S_{x}\left(t\;-\;1\right)H_{k}$ captures both the desired-signal covariance and the multiuser interference seen by the user *k*; the additive noise is modeled by $\sigma^{2}I_{N}$. The matrix inside the inverse is Hermitian positive definite, so Equation (29) is well posed (solved via Cholesky), and $U_{k}^{\left(t\right)}\in C^{N\times d_{k}}$.
- Weight Matrix Update $W_{k}^{\left(t\right)}$: The weight matrix is updated as
  (31) $$W_{k}^{\left(t\right)}=\mu_{k}{\left(E_{k}^{\left(t\right)}+\varepsilon \;I_{d_{k}}\right)}^{-1},\;\forall k.$$
  $W_{k}^{\left(t\right)}=\mu_{k}{\left(E_{k}^{\left(t\right)}+\varepsilon \;I_{d_{k}}\right)}^{-1}\in C^{d_{k}\times d_{k}}$, where $E_{k}^{\left(t\right)}$ is the $d_{k}\times d_{k}$ MSE matrix evaluated with $U_{k}\left(t\right)$ and $P_{k}\left(t\;-\;1\right)$. The small ε>0 regularizes the inversion and improves conditioning, and $\mu_{k}>0$ sets stream/user priorities (e.g., for WSR maximization). Thus $W_{k}^{\left(t\right)}$ is diagonal and positive definite, which is subsequently exploited by our low-complexity update in Equation (32).
- Transmit Precoder Update $P^{\left(t\right)}$: The transmit precoders are updated by solving a convex quadratic problem
  (32) $$P^{\left(t\right)}=\frac{1}{\sigma^{2}}\;\left[B^{\left(t\right)}-H{\left({\left(S^{\left(t\right)}\right)}^{-1}+\frac{1}{\sigma^{2}}H^{H}H\right)}^{-1}\frac{1}{\sigma^{2}}H^{H}B^{\left(t\right)}\right]\in C^{M\times D}.$$
  With $D_{k}^{\left(t\right)}$, $S^{\left(t\right)}$, H, and $B^{\left(t\right)}$ defined in Equations (20)–(23), the Woodbury update in Equation (32) computes the precoder as $P\left(t\right)=\sigma^{-2}\;\left[B^{\left(t\right)}-H{\left({\left(S^{\left(t\right)}\right)}^{-1}+\sigma^{-2}H^{H}H\right)}^{-1}\sigma^{-2}H^{H}B^{\left(t\right)}\right]$, which moves the inversion from size *M* to size NK, yielding per-iteration cost $O\;\left(M{\left(NK\right)}^{2}+{\left(NK\right)}^{3}\right)$ instead of $O(M^{3})$.

**Algorithm 1** Low-Complexity WMMSE (LC-WMMSE) Precoding**Require:** Channel matrices ${\left\{H_{k}\right\}}_{k=1}^{K}$, weights ${\left\{\mu_{k}\right\}}_{k=1}^{K}$, noise $\sigma^{2}$, sum power $P_{\max}$, max iters *T*, tolerance ε  1:Initialize $P^{\left(0\right)}$ s.t. $\sum_{k=1}^{K}{∥P_{k}^{\left(0\right)}∥}_{F}^{2}\leq P_{\max}$  2:**for** t=1 **to** *T* **do**  3:    **for** k=1 **to** *K* **do**  4:        Update receive filter $U_{k}^{\left(t\right)}$ by (29),  5:        Form MSE $E_{k}^{\left(t\right)}$ from $U_{k}^{\left(t\right)}$ and $P^{\left(t-1\right)}$  6:        Update weight $W_{k}^{\left(t\right)}$ by (31),  7:    **end for**  8:    Compute switching factor $\omega^{\left(t\right)}$ by (18) and (19),  9:    **Classical candidate:** build A,B and compute $P_{\mathrm{WMMSE}}^{\left(t\right)}$ by (13)–(15),10:    **Low-complexity candidate:** compute $P_{\mathrm{LC}}^{\left(t\right)}$ by (20)–(23),11:    Hybrid precoder update by (17),12:    Compute damping $\alpha^{\left(t\right)}$ by (25),13:    Damped update $P^{\left(t\right)}$ by (26),14:    Power normalization via (24),15:    Compute ${WSR}^{\left(t\right)}$ by (27),16:    **if** $|WSR^{\left(t\right)}-WSR^{\left(t-1\right)}|<\varepsilon$ **then**17:        **break**18:    **end if**19:**end for****Ensure:** $P^{\left(t\right)}=\left[P_{1}^{\left(t\right)},\ldots ,P_{K}^{\left(t\right)}\right]$

### 3.4. Convergence Analysis

The classical WMMSE algorithm alternates minimization of a convex quadratic surrogate, guaranteeing monotonic ascent of the weighted sum-rate (WSR) [21]. In our LC-WMMSE variant, at iteration *t*, we compute the low-complexity update ${\hat{P}}^{\left(t\right)}$ by solving the diagonal-weighted surrogate problem (replacing $W_{k}$ with $D_{k}=\mathrm{diag}\left(W_{k}\right)$), then apply the damped update:

(33) $$P^{\left(t+1\right)}=P^{\left(t\right)}+\alpha^{\left(t\right)}\left({\hat{P}}^{\left(t\right)}-P^{\left(t\right)}\right),\;\alpha^{\left(t\right)}\in \left(0,1\right],$$

followed by sum power constraint normalization. The step size $\alpha^{\left(t\right)}$ is selected via Armijo backtracking to ensure immediate WSR improvement.

**Proposition** **2.** *(Convergence): Under the stated Armijo acceptance rule, the sequence $WSR(P^{\left(t\right)})$ is non-decreasing and converges. We define the transmit-update quadratic at iteration t as $Q^{\left(t\right)}\left(P\right)=\frac{1}{2}\langle P,\;A^{\left(t\right)}P\rangle -ℜ\left\{\langle B^{\left(t\right)},\;P\rangle \right\}$ built from ${\left\{\left(U_{k}^{\left(t\right)},W_{k}^{\left(t\right)}\right)\right\}}_{k=1}^{K}$. At each iteration t, choose ${\hat{P}}^{\left(t\right)}\in \left\{P_{\mathrm{WMMSE}}^{\left(t\right)},{\hat{P}}_{\mathrm{LC}}^{\left(t\right)}\right\}$ that minimizes $Q^{\left(t\right)}$, and set $P^{\left(t+1\right)}=\left(1-\alpha^{\left(t\right)}\right)P^{\left(t\right)}+\alpha^{\left(t\right)}{\hat{P}}^{\left(t\right)}$ with Armijo backtracking on $\alpha^{\left(t\right)}\in \left(0,1\right]$ until $Q^{\left(t\right)}\left(P^{\left(t+1\right)}\right)\leq Q^{\left(t\right)}\left(P^{\left(t\right)}\right)-\gamma \;\alpha^{\left(t\right)}{∥{\hat{P}}^{\left(t\right)}-P^{\left(t\right)}∥}_{F}^{2},$ for some γ>0. Then, the sequence $\left\{Q^{\left(t\right)}\left(P^{\left(t\right)}\right)\right\}$ is non-increasing and convergent. Any limit point of $\left(U_{k}^{\left(t\right)},W_{k}^{\left(t\right)},P^{\left(t\right)}\right)$ is a stationary point of the classical WMMSE objective if the diagonal surrogate error vanishes asymptotically (i.e., $W_{k}^{\left(t\right)}$ becomes diagonally dominant or the hybrid selection converges to $P_{\mathrm{WMMSE}}^{\left(t\right)}$). Otherwise, the limit point is stationary for the surrogate objective with $D_{k}=\mathrm{diag}\left(\mathrm{diag}\left(W_{k}\right)\right)$.*

**Proof.**  Non-decrease is followed by construction of the acceptance rule. The WSR objective is bounded above under finite SNR and an SPC. Thus $WSR(P^{\left(t\right)})$ is a bounded, non-decreasing sequence and therefore converges. Hybrid selection ensures the best descent direction for $Q^{\left(t\right)}$; Armijo backtracking gives sufficient decrease, and boundedness below implies convergence of $Q^{\left(t\right)}$. The overall scheme is an inexact block-coordinate method; standard results yield stationarity of limit points under vanishing inexactness.    □

### 3.5. Computational Complexity Analysis

Computational complexity is critical for evaluating precoding algorithms in massive MU-MIMO systems. In the classical WMMSE update [21], the dominant per-iteration cost is the M×M precoder solve Equation (15), namely the factorization of $\sigma^{2}I_{M}+\sum_{k=1}^{K}H_{k}U_{k}W_{k}U_{k}^{H}H_{k}^{H}$, which is $O(M^{3})$. Receiver and weight updates each cost $O(KN^{3})$. The R-WMMSE algorithm [10], which has linear complexity of O(M).

Using the LC-WMMSE (Woodbury) identity, we rewrite

(34) $$A^{-1}={\left(\sigma^{2}I_{M}+HSH^{H}\right)}^{-1}=\frac{1}{\sigma^{2}}\;\left[I_{M}-H{\left(S^{-1}+\frac{1}{\sigma^{2}}H^{H}H\right)}^{-1}H^{H}/\sigma^{2}\right],$$

With $H=\left[H_{1},\ldots ,H_{K}\right]\in C^{M\times NK}$ and $S=\mathrm{blkdiag}(S_{1},\ldots ,S_{K})$, $S_{k}=U_{k}\;\mathrm{diag}\left(W_{k}\right)\;U_{k}^{H}\in C^{N\times N}$. The dominant costs per iteration are

- Cholesky/solve of $T=S^{-1}+\frac{1}{\sigma^{2}}H^{H}H\in C^{\left(NK)\times (NK\right)}$: $O\;\left({\left(NK\right)}^{3}\right)$.
- Gram products and multiplies with H (e.g., $H^{H}H$, $H^{H}B$): $O\;\left(M{\left(NK\right)}^{2}\right)$.
- Per-user N×N factorizations (for $S_{k}^{-1}$ and $U_{k}$): $O(KN^{3})$.

Algorithm 1 has a dominant per-iteration cost $O\;\left(M{\left(NK\right)}^{2}\right)+O\;\left({\left(NK\right)}^{3}\right)+O\left(KN^{3}\right)$. In the massive-MIMO regime M≫NK, this is much smaller than $O(M^{3})$. Computing the hybrid switching factor $\omega^{\left(t\right)}$ uses Frobenius norms of N×N MSE matrices, costing $O(KN^{2})$; the damping $\alpha^{\left(t\right)}$ is a few scalar operations, O(1). Both mechanisms reduce the total number of iterations *T*, further lowering wall-clock time. Table 4 summarizes the per-iteration computational complexity of each component for classical WMMSE versus the proposed LC-WMMSE (Woodbury) implementation.

Takeaway: because NK≪M in massive MU-MIMO, LC-WMMSE replaces the cubic $O(M^{3})$ term with operations that scale with NK, yielding the speedups observed in Section 4 while preserving WSR performance.

### 3.6. Implementation Considerations and Overhead Analysis

At iteration *t*, the BS requires downlink (DL) channel state information (CSI) $H_{k}$ and the current receive filters $U_{k}^{\left(t\right)}$ and weights $W_{k}^{\left(t\right)}$. In Time Division Duplex (TDD), the BS estimates $H_{k}$ from Uplink (UL) pilots and computes $U_{k}^{\left(t\right)}$, $W_{k}^{\left(t\right)}$ locally (no DL feedback per iteration). In Frequency Division Duplex (FDD), user equipments (UEs) estimate from DL pilots and feed back either (i) full $U_{k}^{\left(t\right)}\;\in \;C^{N\times d_{k}}$ and Hermitian $W_{k}^{\left(t\right)}\;\in \;C^{d_{k}\times d_{k}}$, or (ii) an LC mode with only $\mathrm{diag}\left(W_{k}^{\left(t\right)}\right)\;\in \;R^{d_{k}}$ plus a compressed $U_{k}^{\left(t\right)}$ (e.g., codebook index). With $b_{c}$ bits per complex and $b_{r}$ per real, the per-user payload is $\approx Nd_{k}b_{c}+\frac{d_{k}\left(d_{k}+1\right)}{2}b_{r}$ (full) vs. $d_{k}b_{r}$ + codebook bits (LC).

## 4. Simulations and Results

### 4.1. Simulation Setup

We consider a single-cell massive MU-MIMO downlink system, where the BS is equipped with *M* transmit antennas and serves *K* users, and each user receives a number of data streams equal to their number of receive antennas, with $d_{k}=N$. The total sum power of the BS under the SPC case is set to be $P_{\max}=10\;\left[W\right]$. The channel matrix H is generated according to a circularly symmetric standard complex normal distribution with pathloss between the users and the BS. The pathloss model is set to be $128.1+37.6\log_{10}\left(d\right)\;\left[\mathrm{dB}\right]$ [26], where *d* denotes the distance between the user and the BS taking range in [0.1∼0.3]km. The noise power is set to be equal for all users and is given by $\sigma^{2}=\frac{P_{max}}{10^{SNR/10}}$, where the signal-to-noise ratio (SNR) is the average received SNR for all users when no precoding is used. For all simulations, we use the hybrid switching constant $\kappa =10^{-3}$, damping scale $\eta =10^{-3}$, damping bounds $\alpha_{\min}=0.2$ and $\alpha_{\max}=0.9$, convergence tolerance $\epsilon =10^{-4}$, and maximum iterations T=100. Our simulation results are averaged over 100 randomly generated channel realizations and are conducted under the assumption of perfect channel state information (CSI) at the base station. All computations are performed using an intel i7-12700H with RTX Graphics, 3.20 GHz CPU, 16 GB RAM, Windows 11 (64-bit) operating system, and Matlab R2024b environment.

### 4.2. Low-Complexity (LC WMMSE)

In this subsection, we provide simulation results evaluating the performance of the proposed LC-WMMSE algorithm with hybrid switching and adaptive damping. We compare our method with other baselines, including the WMMSE algorithm in [21] and the R-WMMSE algorithm [10], the non-iterative baseline precoding methods such as the ZF precoding $P_{\mathrm{ZF}}=H^{H}{\left(HH^{H}\right)}^{-1}$ [27], and the BD precoding $P_{k}=V_{E,0}T_{k}$ [28]. These closed-form methods leverage low-dimensional channel properties (e.g., BD uses null-space projection for interference suppression) and offer low computational complexity ZF: $O(K^{3})$, BD: $O(KM^{2})$ [27,28], making them practical for massive MU-MIMO systems despite their suboptimal WSR performance. For each trial, we draw $P^{\left(0\right)}∼\mathrm{CN}\left(0,1\right)$ and scale to satisfy the same $P^{\left(0\right)}$ is used for all methods to ensure fairness.

**Figure 2:**
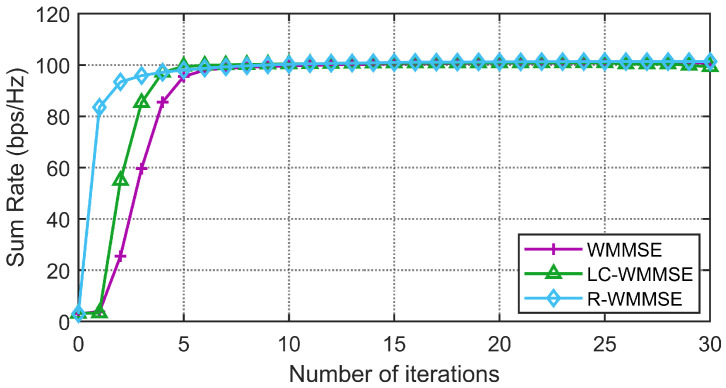
(M=64, K=12, N=2, 10 dB) Convergence of the proposed LC-WMMSE algorithm and the classical WMMSE algorithm.

**Figure 3:**
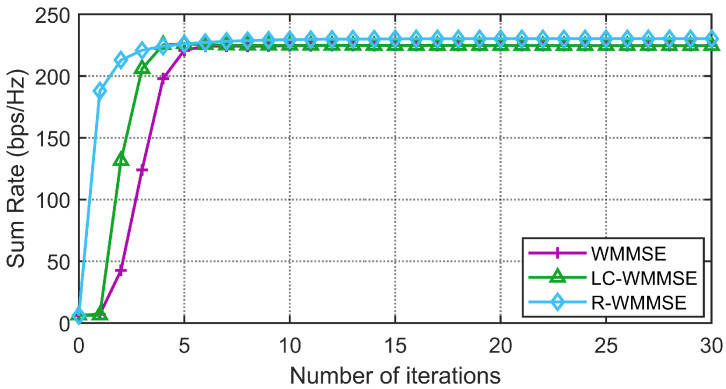
(M=128, K=16, N=4, 0 dB) Convergence of the proposed LC-WMMSE algorithm and the classical WMMSE algorithm.

First, we show the convergence performance of the proposed LC-WMMSE algorithm and the classical WMMSE algorithm in Figure 2 and Figure 3. The WSR is measured by bits per second per hertz (bps/Hz). Figure 2 and Figure 3 clearly show the proposed LC-WMMSE algorithm and the WMMSE algorithm converge to the same WSR value. Furthermore, it is observed that starting from the same initial point, the LC-WMMSE algorithm often achieves faster convergence in the initial iterations compared to the WMMSE algorithm, while also maintaining competitive performance with the state-of-the-art R-WMMSE algorithm [10] which employs randomized approximations for complexity reduction.

**Figure 4:**
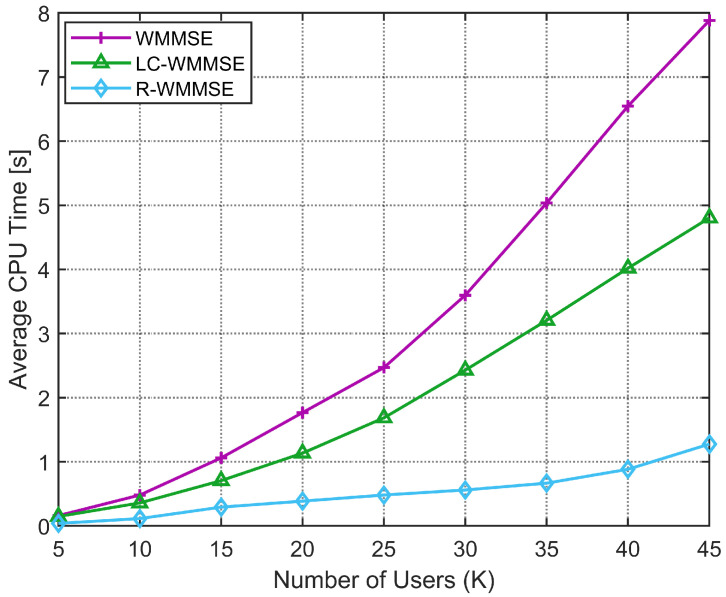
Average CPU time to convergence versus number of users *K* (M=128, N=2, 10 dB).

Secondly, we compare the proposed LC-WMMSE algorithm with the WMMSE algorithm and R-WMMSE algorithm in terms of the average CPU execution time to convergence under different numbers of users *K* and different numbers of BS transmit antennas *M*. As can be seen in Figure 4, comparing the average computational complexity, measured in CPU execution time, of the classical WMMSE algorithm and our proposed LC-WMMSE algorithm. The simulation considers a scenario with M=128 BS antennas, N=2 receive antennas per user, and an average SNR of 10 dB. When the number of users *K* increases, both algorithms show rising computational demands. While runtime increases with *K* for all methods, LC-WMMSE exhibits a noticeably flatter growth than classical WMMSE, reflecting its lower per-iteration cost. R-WMMSE—using randomized/sketched updates—achieves the shortest times overall. At K=40, LC-WMMSE requires 4s and 6.7s for WMMSE (≈40% reduction), and the R-WMMSE completes in about 0.9s, i.e., ≈86% faster than WMMSE. However, the proposed LC-WMMSE algorithm consistently achieves lower complexity than the classical WMMSE algorithm.

**Figure 5:**
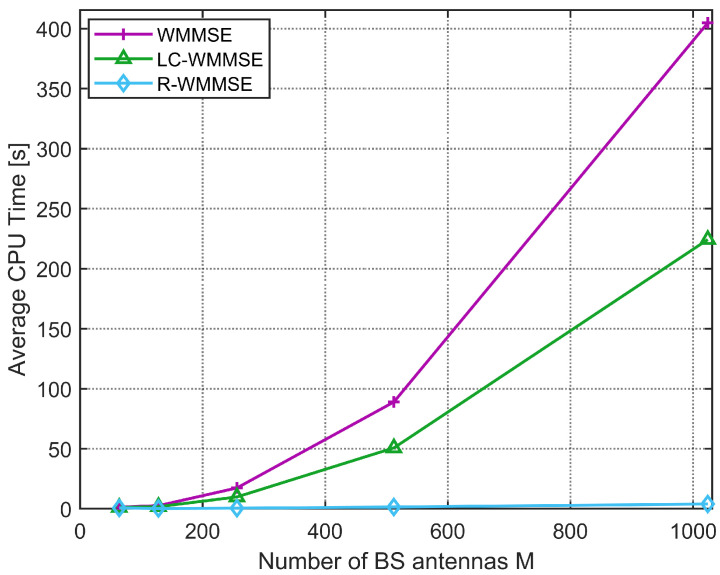
Average CPU time to convergence versus the number of BS antennas *M* (K=16, N=4, 10 dB).

This demonstrates the efficiency of our proposed algorithm, highlighting its suitability for massive MU-MIMO systems, where the number of supported users is typically high. As shown in Figure 5, the simulation scenario is configured with K=16 users, each user with N=4 receive antennas, at an average SNR of 10 dB. It can be observed that the computational cost for both algorithms increases with *M*. Especially when M=1024, the classical WMMSE algorithm will take 410 s to converge, while our proposed LC-WMMSE algorithm takes 225 s and the R-WMMSE algorithm takes 4 s because the R-WMMSE algorithm has linear complexity O(M). For instance, at M>1000, the LC-WMMSE algorithm achieves a 1.8× speedup over the classical WMMSE algorithm method. The simulation results presented clearly validate our complexity analysis, demonstrating that the LC-WMMSE algorithm achieves low complexity scaling with respect to *M*, whereas the classical WMMSE exhibits cubic complexity.

**Figure 6:**
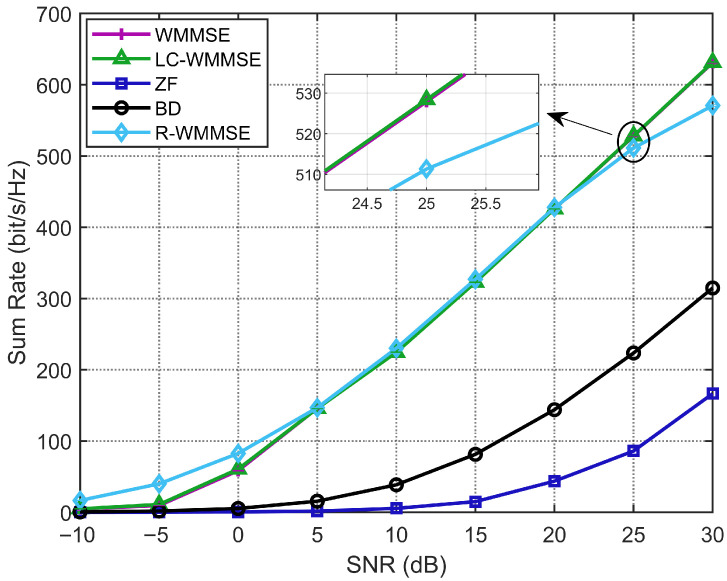
Weighted sum-rate performance with different SNRs (M=128, K=16, N=4).

Lastly, we show the WSR performance of our proposed LC-WMMSE algorithm and other baselines with SNR under the set: M=128, K=16, and N=4. As shown in Figure 6, our proposed LC-WMMSE algorithm achieves almost the same performance as the classical WMMSE algorithm, and the R-WMMSE algorithm yields almost the same performance as the LC-WMMSE algorithm but significantly outperforms the BD and ZF algorithms under different SNR values. Over 0–30 dB, the mean relative gap $\Delta_{\mathrm{rel}}$ of LC-WMMSE to WMMSE is −0.44% for i.i.d. Rayleigh (see Table 5).

### 4.3. Performance Under Correlated Channels

We also assess robustness under spatial correlation using a Kronecker model at the BS array. For the user *k* the channel is

(35) $$H_{k}\;=\;R_{\mathrm{BS}}^{1/2}\;W_{k},\;W_{k}∼\mathrm{CN}\;\left(0,\;I_{M\times N}\right),$$

With exponential BS correlation,

(36) $${\left[R_{\mathrm{BS}}\right]}_{m,n}=r^{\left|m-n\right|},\;0\leq r<1.$$

All experiments we set r=0.5, r=0.7 and obtain $R_{\mathrm{BS}}^{1/2}$ from the Hermitian eigendecomposition of $R_{\mathrm{BS}}$. We symmetrize $R_{\mathrm{BS}}$ numerically and clip tiny negative eigenvalues before taking square roots. Unless noted, the simulation protocol (SNR grid, (M,N,K), initialization, tolerances, and power normalization) is identical to the i.i.d. case.

**Figure 7:**
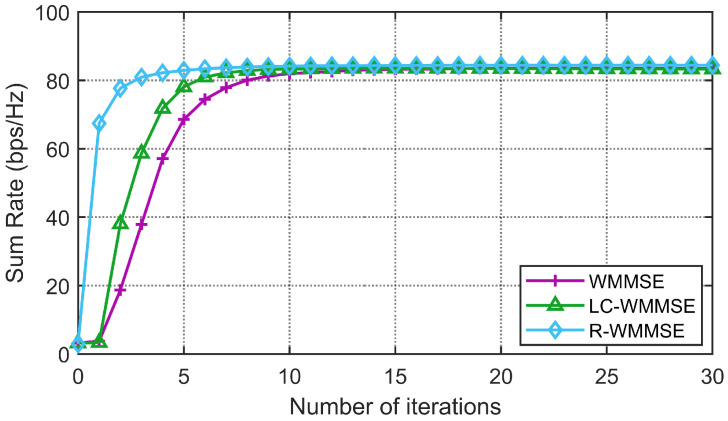
Correlated r = 0.7 (M = 64, K = 12, N = 2, 10 dB), convergence of the proposed LC-WMMSE algorithm and the classical WMMSE algorithm.

**Figure 8:**
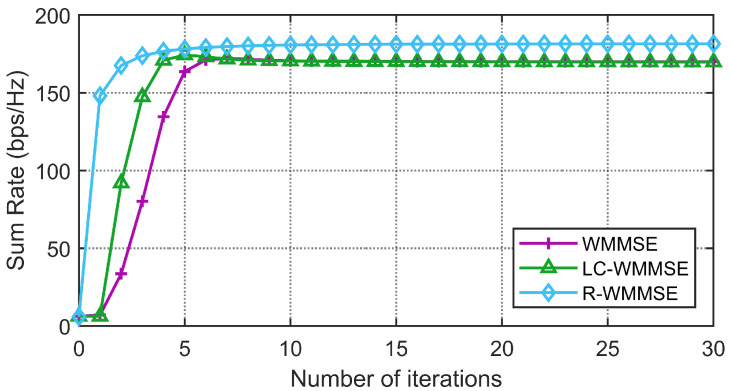
Correlated r = 0.7 (M = 128, K = 16, N = 4, 0 dB), convergence of the proposed LC-WMMSE algorithm and the classical WMMSE algorithm.

Figure 7 and Figure 8 demonstrate convergence behavior under a Kronecker-correlated channel with r=0.7 for two system sizes and SNRs (means over 100 trials). In both scenarios, LC-WMMSE closely tracks classical WMMSE and reaches the same final WSR, while R-WMMSE converges fastest to a similar value. Over the 0–30 dB practical regime, the mean LC-WMMSE gap under correlation is approximately 0.5% (see Table 5). The effect of strong correlation is mainly visible in the early-iteration transient; the steady-state WSR gap between LC-WMMSE and WMMSE remains negligible, confirming robustness across scales and SNR.

**Figure 9:**
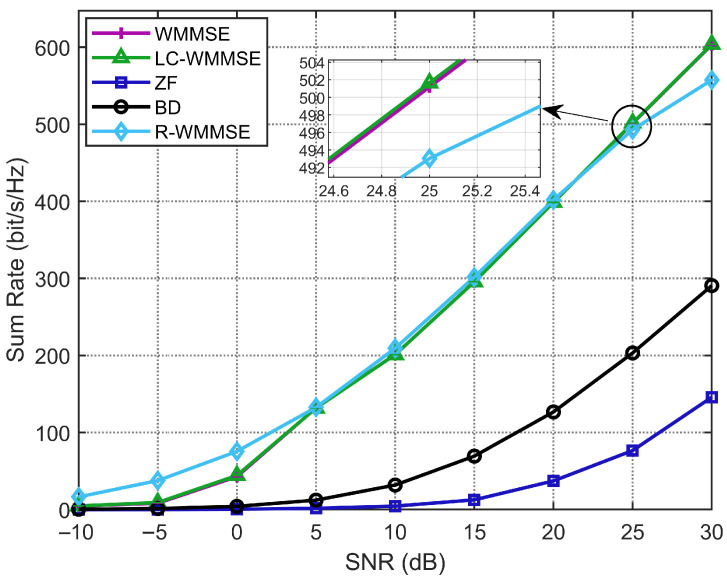
Weighted sum-rate performance with different SNRs under correlated channels (M=128, K=16, N=4).

Figure 9 reports the weighted sum-rate (WSR) versus SNR under BS correlation. As expected, all methods degrade relative to i.i.d. Rayleigh due to reduced spatial degrees of freedom. Importantly, the proposed LC-WMMSE closely tracks classical WMMSE across the entire SNR range while preserving the computational gains reported earlier. R-WMMSE remains the fastest baseline, and the gap between LC-WMMSE and classical WMMSE is visually negligible in WSR, consistent with the i.i.d. case. Over 0–30 dB, the mean relative gap $\Delta_{\mathrm{rel}}$ of LC-WMMSE to WMMSE is −0.48% for correlated channels (see Table 5). Notably, Table 6 reveals a degradation of R-WMMSE at 30 dB for correlated channels. The cause is sketch-induced approximation bias in solving ill-conditioned normal equations at high SNR; in contrast, deterministic WMMSE and LC-WMMSE avoid this issue and retain the higher WSR.

**Remark** **2.** *(UE correlation): The same framework accommodates UE-side correlation by using $H_{k}=R_{\mathrm{BS}}^{1/2}\;W_{k}\;R_{\mathrm{UE}}^{1/2}$ with, e.g., ${\left[R_{\mathrm{UE}}\right]}_{m,n}=r_{\mathrm{UE}}^{\left|m-n\right|}$.*

## 5. Conclusions

Weighted sum-rate (WSR) maximization is a fundamental problem for massive MU-MIMO systems. This article has investigated the WSR maximization problems of massive MU-MIMO systems. We introduced a novel LC-WMMSE precoding algorithm specifically designed for massive MU-MIMO downlink systems. To significantly reduce the computational runtime with the classical WMMSE precoding method, our approach integrates a hybrid switching mechanism and an adaptive damping strategy. The core innovation employs the Woodbury matrix identity to transform the dominant $O(M^{3})$ matrix inversion into smaller $O({\left(NK\right)}^{3})$ operations, while the hybrid switching dynamically balances the computationally intensive standard WMMSE updates with simpler approximations, controlled by an adaptive mixing factor. Simultaneously, the adaptive damping mechanism ensures stable and monotonic convergence behavior throughout the iterations. Our simulation results show that the LC-WMMSE algorithm significantly reduces practical runtime while maintaining high WSR performance, making it practical for massive MU-MIMO systems. Our approach provides a computationally efficient drop-in replacement for classical WMMSE, achieving near-identical performance with substantially reduced complexity. The LC-WMMSE update also extends to hybrid beamforming architectures via the effective channel ${\tilde{H}}_{k}=H_{k}F_{A}$ with $N\leftarrow N_{\mathrm{RF}}$; a comprehensive study of hybrid beamforming (incorporating phase constraints, codebooks, and quantization) is deferred to future work. For imperfect CSI, the key challenge is preserving our low-complexity Woodbury/diagonal structure; we will use stochastic/robust WMMSE with diagonal inflations and light Tikhonov regularization so the transmit update remains an (NK)×(NK) SPD solve. For per-antenna power constraints (PAPC), coupling across antennas breaks simple normalization; we will introduce per-antenna dual variables so the update becomes ${\left(A+\mathrm{diag}\;\lambda \right)}^{-1}B$ and compute λ via bisection/ADMM, preserving LC complexity.

## Figures and Tables

**Table 1:** R-WMMSE and LC-WMMSE SPD: symmetric positive definite.

	R-WMMSE	LC-WMMSE (Proposed)
Principle	Randomized sketching	Structure exploitation (Woodbury + diag(W))
Update size	Compressed (by sketch)	(NK)×(NK) SPD solve
Dominant cost	Sketch products + small solve	Build A,B + SPD solve
Error source	Sketching bias/variance	Neglect of off-diagonals in $W_{k}$

**Table 2:** Summary of notations. Bold denotes matrices/vectors, Hermitian transpose, ${\left(\cdot \right)}^{H}$ and Frobenius norm ${∥\cdot ∥}_{F}$.

Notation	Meaning
*M*	Number of BS transmit antennas
*N*	Number of receive antennas per user
*K*	Number of users
$d_{k}$	Number of streams for user *k*
$D≜\sum_{k=1}^{K}d_{k}$	Total number of streams
$H_{k}\in C^{M\times N}$	Channel from BS to user *k*
$P_{k}\in C^{M\times d_{k}}$	Precoder for user *k*
$P=\left[P_{1},\ldots ,P_{K}\right]\in C^{M\times D}$	Stacked precoder (all users)
$s_{k}\in C^{d_{k}\times 1}$	Data vector for user *k*
$x\in C^{M\times 1}$	Transmit signal
$y_{k}\in C^{N\times 1}$	Received signal at user *k*
$n_{k}\in C^{N\times 1}$	AWGN at user *k*
$\sigma^{2}$	Noise power (per receive antenna)
$P_{\max}$	Total transmit power (SPC)
$U_{k}\in C^{N\times d_{k}}$	MMSE receive filter for the user *k*
$W_{k}\in C^{d_{k}\times d_{k}}$	Weight matrix for the *k*-th user
$E_{k}\in C^{d_{k}\times d_{k}}$	MSE matrix for user *k*
$D_{k}\;=\;\mathrm{diag}\left(\mathrm{diag}\left(W_{k}\right)\right)$	Diagonal weight approximation
$S_{x}=\sum_{j=1}^{K}P_{j}P_{j}^{H}\in C^{M\times M}$	BS transmit covariance
$H=\left[H_{1},\ldots ,H_{K}\right]\in C^{M\times (NK)}$	Stacked channel
$S=\mathrm{blkdiag}\left(U_{1}D_{1}U_{1}^{H},\ldots ,U_{K}D_{K}U_{K}^{H}\right)\in C^{NK\times NK}$	Block diag. weight (LC)
$B_{\mathrm{LC}}=\left[\;H_{1}U_{1}D_{1},\;\ldots \;,H_{K}U_{K}D_{K}\;\right]\in C^{M\times D}$	RHS factor (LC update)
$B_{\mathrm{class}}=\left[\;H_{1}U_{1}W_{1},\;\ldots \;,H_{K}U_{K}W_{K}\;\right]\in C^{M\times D}$	RHS factor (classical)
$G=H^{H}H\in C^{\left(NK)\times (NK\right)}$	Stacked Gram matrix
$I_{M},\;I_{N},\;I_{d_{k}}$	Identity matrices of sizes *M*, *N*, $d_{k}$
diag(·),blkdiag(·),tr(·)	Standard operators
$P_{\mathrm{WMMSE}}^{\left(t\right)}$	Classical WMMSE precoder (iter. *t*)
$P_{\mathrm{LC}}^{\left(t\right)}$	LC–WMMSE precoder (iter. *t*)
$\omega^{\left(t\right)}$	Hybrid switching factor (iter. *t*)
$\alpha^{\left(t\right)}$	Adaptive damping factor (iter. *t*)
$WSR^{\left(t\right)}$	Weighted sum-rate at iter. *t* (bps/Hz)

**Table 3:** Ablation study of damping mechanisms (mean over 100 trials at 20 dB).

Metric	Adaptive	Fixed (α=0.8)	None
Final WSR [bit/s/Hz]	424.90±2.01	425.79±2.03	424.42±2.12
Iterations (median)	50	50	50
Oscillation index	0.067	0.012	0.101

**Table 4:** Per-iteration computational complexity.

Operation	Classical WMMSE	LC–WMMSE (Woodbury)
Precoders solve	$O(M^{3})$	$O\;\left(M{\left(NK\right)}^{2}\right)+O\;\left({\left(NK\right)}^{3}\right)$
Per-user N×N factorizations	$O(KN^{3})$	$O(KN^{3})$
Gram products ($H^{H}H$, $H^{H}B$)	$O\;\left(M{\left(NK\right)}^{2}\right)$	$O\;\left(M{\left(NK\right)}^{2}\right)$
Hybrid switch $\omega^{\left(t\right)}$	–	$O(KN^{2})$
Adaptive damping $\alpha^{\left(t\right)}$	–	O(1)
Total (dominant)	$O(M^{3}+KN^{3})$	$O\;\left(M{\left(NK\right)}^{2}+{\left(NK\right)}^{3}+KN^{3}\right)$

**Table 5:** Practical regime (0–30 dB) summary of the relative WSR gap $\Delta_{\mathrm{rel}}\left(\%\right)=100\left({\mathrm{WSR}}_{\mathrm{WMMSE}}-{\mathrm{WSR}}_{\mathrm{LC}}\right)/{\mathrm{WSR}}_{\mathrm{WMMSE}}$. Values are mean and std across SNRs; worst-loss is the maximum positive gap; best-gain is the maximum $-\min\Delta_{\mathrm{rel}}$ (LC over WMMSE). A negative mean indicates LC-WMMSE exceeds classical WMMSE. Setup: M=128, N=4, K=16, SPC $P_{max}\;=\;10$, 100 trials/SNR.

Model	Mean ↓	Std	Worst-Loss ↓	Best-Gain ↑
i.i.d. Rayleigh	−0.440	0.294	0.234	4.843
Correlated (α=0.5)	−0.480	0.371	0.218	6.529

**Table 6:** Weighted sum -rate (bits/s/Hz) comparison under spatial correlation conditions.

Algorithm	Moderate Correlation (r = 0.5)			Strong Correlation (r = 0.7)		
	10 dB	20 dB	30 dB	10 dB	20 dB	30 dB
WMMSE	201	398	603	170	359	563
LC-WMMSE	201	399	604	170	359	564
R-WMMSE	209	401	557	181	364	536
ZF	5	37	146	3	28	130
BD	32	127	290	23	103	258

## Data Availability

Data are contained within the article.
